# Superior colliculus neurons encode a visual saliency map during free viewing of natural dynamic video

**DOI:** 10.1038/ncomms14263

**Published:** 2017-01-24

**Authors:** Brian J. White, David J. Berg, Janis Y. Kan, Robert A. Marino, Laurent Itti, Douglas P. Munoz

**Affiliations:** 1 Centre for Neuroscience Studies, Queen's University, 18 Stuart St, Kingston, Ontario, Canada K7L3N6; 2 IBM Research, Almaden, 650 Harry Road, San Jose, California 95120, USA; 3 Department of Computer Science, University of Southern California, 0781, 941 Bloom Walk, Los Angeles, California 90089, USA

## Abstract

Models of visual attention postulate the existence of a saliency map whose function is to guide attention and gaze to the most conspicuous regions in a visual scene. Although cortical representations of saliency have been reported, there is mounting evidence for a subcortical saliency mechanism, which pre-dates the evolution of neocortex. Here, we conduct a strong test of the saliency hypothesis by comparing the output of a well-established computational saliency model with the activation of neurons in the primate superior colliculus (SC), a midbrain structure associated with attention and gaze, while monkeys watched video of natural scenes. We find that the activity of SC superficial visual-layer neurons (SCs), specifically, is well-predicted by the model. This saliency representation is unlikely to be inherited from fronto-parietal cortices, which do not project to SCs, but may be computed in SCs and relayed to other areas via tectothalamic pathways.

## Abstract (web summary)

Saliency maps have been proposed to guide visual attention, yet the underlying neural correlates remain undetermined. Here, the authors record from monkeys as they watch videos of natural scenes, and find superior colliculus superficial visual-layer neurons exhibit activity patterns consistent with a visual saliency map.

Since its introduction almost 30 years ago^1^, saliency-map theory (Fig. 1a) has attracted wide-spread attention^2^, with an explosion of applications not only in neuroscience and psychology, but also in machine vision, surveillance, defence, transportation, medical diagnosis, design and advertising. The concept of a priority map^3^,^4^ (Fig. 1a, blue) arose as an extension of this idea to include top-down, goal-dependent input in a combined representation of visual saliency and behavioural relevancy, which is thought to determine attention and gaze.

Empirical and computational modelling studies have reported evidence of a saliency and/or priority map in various cortical brain areas (for example, V1 (refs 5, 6, 7); V4 (refs 8, 9); lateral intraparietal area, LIP^10^,^11^,^12^; frontal eye fields, FEF^13^; dorsolateral prefrontal cortex^14^). While these studies used intuitive definitions of saliency with simple stimuli under restricted viewing conditions, a strong test of the saliency hypothesis would be to use a well-established computational model to correlate neuronal firing rates with model-predicted saliency during unconstrained viewing of natural dynamic scenes. This requires that saliency be quantitatively defined at every location and time in any stimulus stream, which is best achieved with a computational saliency model^1^,^15^,^16^.

There is evidence for a subcortical saliency mechanism in the avian optic tectum^17^,^18^,^19^, which pre-dates the evolution of neocortex. The superior colliculus^20^ (SC; the mammalian homologue of the optic tectum) is a multilayered midbrain structure with two dominant functional layers ideally suited for the role of a saliency versus priority map, a visual-only superficial layer (SCs), and a multisensory, cognitive, motor-related intermediate layer (SCi) (Fig. 1b). We hypothesize that visual saliency is coded in the primate SCs (Fig. 1b) because: (1) it is heavily interconnected with early visual areas^20^; (2) it encodes stimuli in a featureless manner^21^,^22^,^23^; (3) it has a well-defined topography^24^; and (4) it has long-range centre-surround organization well suited for a saliency mechanism^25^. In contrast, we hypothesize that visual saliency is poorly represented in the SCi because activation of these neurons is highly dependent upon goal-directed attention and gaze behaviour^26^,^27^,^28^,^29^, owing to its dominant inputs from frontal/parietal areas and basal ganglia, and its direct output to the brainstem saccade circuit^30^. This has led to the hypothesis that SCi best represents a behavioural priority map^4^.

## Results

### Saliency maps to dynamic scenes in monkey SC

To test the hypothesis that SCs represents a visual saliency map, three rhesus monkeys freely viewed a series of high-definition video clips of natural dynamic scenes while we recorded extracellular activity of single SC neurons (Fig. 2a). Saliency maps were generated by a computational saliency model that was (i) built from the architecture of earlier, now well-established, models^1^,^15^,^16^, (ii) validated on the viewing behaviour of rhesus monkeys^31^, (iii) coarsely tuned to the primate visual and oculomotor systems^32^ and (iv) computed in gaze-centred log-polar space corresponding to the SC maps^24^ (Fig. 2b,c; see Methods).

Although there were no structured events to time-lock to during free viewing, we defined naturally occurring fixations as events, because they represent the window in which the visual system integrates visual information. Excluding fixations less than 200 ms (see Methods and Supplementary Fig. 1), we obtained between 65 and 1,812 fixations per neuron (*n*=50,659 total fixations over 4,267 clip viewings). The neurons were separated into two main functional types^20^, SCs visual neurons and SCi visuomotor neurons (see Methods), to examine differences in saliency coding between the two brain areas. For each fixation, we computed the average model-predicted saliency within each neuron's receptive field (RF) boundary during a saccade-free period from 0 to 200 ms post-fixation. Figure 2d shows a Spearman correlation between model-predicted saliency and firing rate (spks per s) for an example SCs neuron (each dot represents a single fixation). Seventy-nine percent (27/34) of SCs neurons (Fig. 2e) and 62% (16/26) of SCi neurons (Fig. 2f) showed a significant positive correlation between continuous firing rate and model-predicted saliency. In addition, both distributions of *r*-values (Fig. 2e,f) were shifted significantly to the right of zero indicating overall positive correlations (*P*=4.1e−06 for SCs, *P*=2.4e−05 for SCi, Wilcoxon sign-rank test).

Fixations were then sorted and binned into tertiles of saliency level in the RF (low, medium, high). For each neuron, we plotted the averaged firing rate at each saliency level as a function of time from fixation. Figure 2 shows four example neurons, two from SCs (Fig. 2g,h) and two from SCi (Fig. 2j,k). The example SCs neurons showed a clear systematic increase in post-fixation firing rate with each increment in model-predicted saliency. This pattern of results was significant in the population average of SCs neurons (Fig. 2i; *F*(2, 66)=35.69*, P*=3.12e−11, repeated-measures ANOVA). This trend was similar for SCi but overall weaker, as illustrated by the example SCi neurons (Fig. 2j,k), and the more modest, but significant, trend in the population averages (Fig. 2l; *F*(2, 50)=14.98*, P*=8.01e−06, repeated-measures ANOVA). This overall pattern of results was observed in all three animals (M1: *F*(2, 52)=12.09*, P*=4.86e−05; M2: *F*(2, 22)=34.88*, P*=1.50e−07; M3: *F*(2, 40)=19.97*, P*=9.69e−07).

Next, we asked to what extent this saliency representation was modulated by the behavioural goal of the animal. Theoretically, a saliency map should represent visual conspicuity irrespective of saccade goal^1^,^15^,^16^, in contrast to a priority map, whose output determines the locus of attention and gaze^4^. To address this question, we selected a subset of the total fixations based on the next saccade direction (Saccade-goal in versus Saccade-goal opposite the RF; Fig. 3, top illustration). Figure 3 shows the average normalized firing rate, aligned on fixation onset, as a function of saliency and saccade-goal, for SCs (Fig. 3a,b) and SCi (Fig. 3d,e) neurons, respectively. Figure 3c,f shows normalized firing rate averaged across the saccade-free epoch illustrated by the shading from 0 to 200 ms in Fig. 3a,b,d,e. We ran a three-way mixed ANOVA with saliency (high–low) and saccade-goal (in-opposite) as repeated-measures factors, and neuron-type (SCs–SCi) as an independent-measures factor. The ANOVA revealed a significant three-way interaction (*F*(1, 58)=4.47, *P*=0.039). To simplify the result, we ran a subsequent two-way ANOVA (saliency × saccade-goal) for SCs and SCi neurons, separately. For SCs neurons (Fig. 3c), the interaction was not significant (*F*(1, 33)<1, *P*=0.37), but there was a highly significant main effect of saliency (*F*(1, 33)=62.34, *P*=1.0e−07), and a significant main effect of saccade-goal (*F*(1, 33)=12.58, *P*=0.0011). Thus, the activation of our sample of SCs neurons was well-predicted by the model, and this effect was highly significant irrespective of the goal of the next saccade. In contrast, for SCi neurons (Fig. 3f), the interaction between saliency and saccade-goal was statistically significant (*F*(1, 25)=6.78, *P*=0.015). Subsequent Bonferroni-corrected comparisons revealed that the saliency effect for SCi neurons was present only in the saccade-goal in condition (in: *t*(25)=4.50, *P*=1.36e-04; opposite: *t*(25)=1.44, *P*=0.16; paired *t*-tests, 1-tailed), and most importantly it was markedly weaker than in SCs (*t*(58)=4.37, *P*=2.5432e−05; independent *t*-test, 1-tailed). In addition, the saliency representation emerged later in SCi than SCs (note the tick marks above the abscissa in Fig. 3a,b,d indicating the period at which the response curves diverged).

We ran the same analyses on the data aligned on saccade onset (Fig. 4). Here, averaging was done over a saccade-free epoch from −150 to −50 ms relative to saccade onset (illustrated by the grey shaded region in Fig. 4a,b,d,e. For SCs neurons (Fig. 4c), the interaction was again not significant (*F*(1, 33)=1.1, *P*=0.298), and like the fixation-aligned data, there was a highly significant main effect of saliency (*F*(1, 33)=64.26, *P*=1.0e−07), and a significant main effect of saccade-goal (*F*(1, 33)=17.49, *P*=0.0002). Importantly, although there was a main effect of saccade goal in both the fixation- (Fig. 3) and saccade-aligned (Fig. 4) data, the magnitude of the saliency representation (that is, the ratio of the response to low versus high saliency) in SCs remained constant irrespective of saccade-goal. For SCi neurons in the saccade-aligned data (Fig. 4f), the interaction between saliency and saccade-goal was not significant (*F*(1, 25)=1.61, *P*=0.21), but there was a significant main effect of saliency (*F*(1, 25)=10.41, *P*=0.0034), and a significant main effect of saccade-goal (*F*(1, 25)=9.11, *P*=0.0057). While the interaction was not significant, the main effect of saliency for SCi neurons was clearly not driven by the saccade-goal opposite condition (Fig. 4e,f), which was not significant (*t*(25)=0.60, *P*=0.55), whereas the saccade-goal in condition was significant with Bonferroni correction (*t*(25)=3.21, *P*=0.0037). Taking the fixation- and saccade-aligned results together, it is clear that SCi neurons, in contrast to SCs neurons, showed an overall weaker saliency representation, and a clear absence of saliency coding outside the saccade-goal (Figs 3e and 4e). Thus, the pattern of results for SCi neurons is not supportive of a visual saliency map. Qualitatively similar results were observed using receiver operating characteristic analysis (Supplementary Fig. 2).

These data also provide validation for the model as evidenced by the link with saccade behaviour (Fig. 5). Specifically, there was a greater frequency of saccades directed in the RF when saliency in the RF was high (*n*=2,387) versus low (*n*=2,080) (Fig. 5a, *P*=1.31e−06, Wilcoxon independent-samples test). Likewise, the reaction time of saccades directed in the RF was faster (that is, shorter fixation duration) when saliency in the RF was high (mean=299 ms) versus low (mean=314 ms) (Fig. 5b, *P*=4.17e−06, Wilcoxon independent-samples test). Greater model-predicted saliency also led to earlier neuronal selection time for SCi visuomotor neurons only (Supplementary Fig. 3).

Lastly, we asked to what extent the saliency representation in the SC is feature-agnostic (that is, independent of the features that gave rise to saliency), a hallmark of saliency map theory^1^,^15^,^16^. A detailed analysis of the individual feature maps can be seen in Fig. 6. Each column represents a given neuron, and each row represents a given feature. The intensity of the colour is an index of the neuron's response to that feature. Specifically, it represents the difference in activation evoked by high versus low model-predicted feature-saliency (note the colour scale on the left). Although there was some variation in individual neuron's feature preference, on average SC neurons were activated by all features when the saccade goal was in (Fig. 6a; SCs: *t*(33)>4, *P*<0.001; SCi t(25)>3, *P*<0.05; Bonferroni-corrected paired *t*-tests); that is, they represented integrated visual salience as opposed to being strongly tuned to any one feature. When saccade-goal was opposite (Fig. 6b), SCs neurons were again significantly activated by all features (*t*(33)>3, *P*<0.01), whereas SCi neurons were not (*t*(25)<1.8, *P*>0.5, for all but one feature), again supporting the behavioural dependence of SCi neurons reported earlier. This indicates that the pattern of activation for SCs neurons specifically is consistent with a feature-agnostic, combined-cues saliency map. This is in agreement with previous studies indicating that SC neurons are sensitive to features such as colour and motion, but they are not particularly selective for any specific feature^21^,^22^,^23^. This is also in agreement with a previous study showing that the pattern of free viewing gaze behaviour of monkeys with V1 lesion (but spared retina-to-SC projection) remains significantly guided by most features of the saliency model^32^.

## Discussion

**Figure 1:**
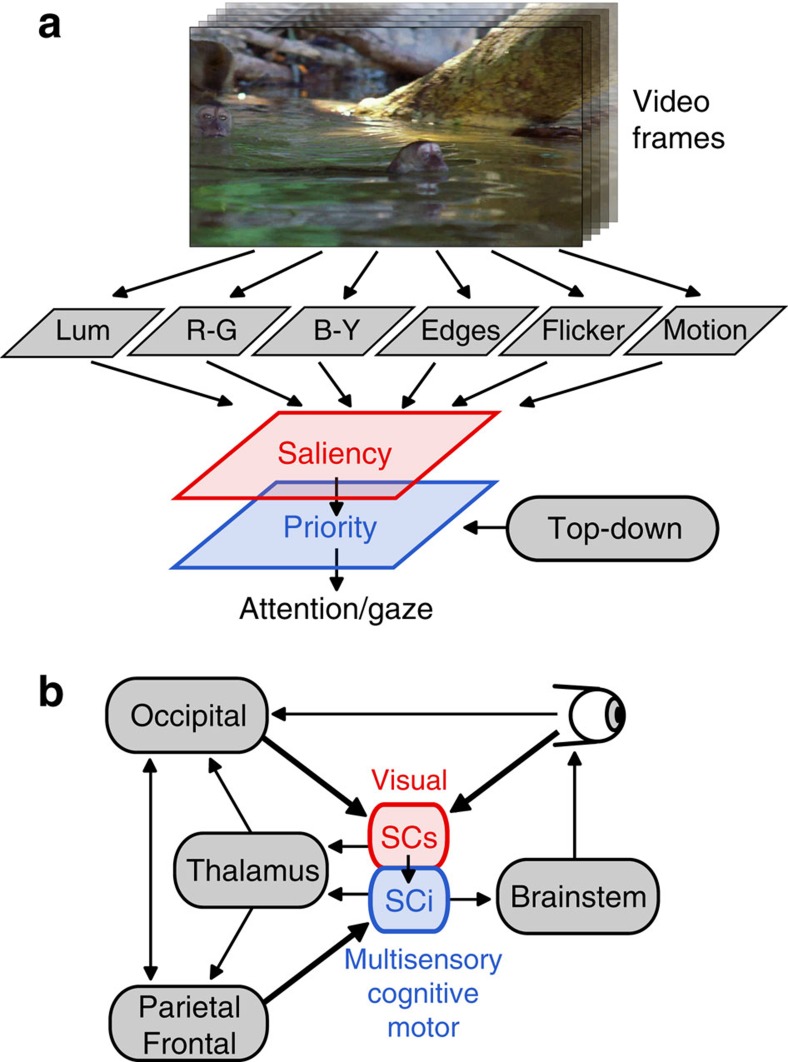
Saliency and priority coding in the superior colliculus (SC). (**a**) Conceptual framework of saliency model. Visual input is decomposed into several topographic feature maps for luminance contrast, colour opponency, oriented edges, flicker and motion. Spatial centre-surround competition for representation in each feature map highlights locations which stand out from their neighbours. All features are integrated into a single saliency map which encodes salience in a feature- and behaviour-agnostic manner and which, combined with top–down signals, gives rise to a priority signal that controls orienting behaviour. Abbreviations: Lum: luminance; R–G: red–green colour opponency; B–Y: blue–yellow colour opponency. (**b**) Simplified schematic of the dominant inputs and outputs of the primate SC. SCs: superior colliculus superficial layers; SCi: superior colliculus intermediate layers.

Here, we showed that SCs neurons, whose dominant inputs arise from the retina and visual cortex^20^ (Fig. 1b), exhibited discharge patterns that were highly consistent with a visual saliency map, when they were recorded during free viewing of natural dynamic scenes. Specifically, SCs neurons showed a reliable correlation between firing rate and the output of a well-established computational saliency model^1^,^15^,^16^, irrespective of the top–down goal of the animal. That is not to say that the response of SCs neurons was not modulated by saccade-goal. Specifically, the magnitude of the saliency representation (that is, the ratio of the response to low versus high saliency) in SCs remained constant independent of saccade-goal (Figs 3c and 4c). Importantly, the activation of SCs neurons was not consistent with a priority map whose peak determines attention and gaze, because peak activation on the SCs map was ambiguous as to the saccade-goal. For example, SCs neurons showed the same or lower peak activation in the saccade-goal in/low-saliency condition (Figs 3a and 4a, light red) as in the saccade-goal opposite/high-saliency condition (Figs 3b and 4b, dark red; see also Figs 3c and 4c comparing the light red symbol in the saccade-goal in condition with dark red symbol in the saccade-goal opposite condition). This indicates that the output of the SCs map alone cannot provide sufficient information to determine gaze, while its output did provide a good index of model-predicted saliency.

SCi neurons, which represent a later sensorimotor processing stage, and receive inputs from fronto-parietal areas^20^ (Fig. 1b), showed a clear absence of a saliency representation outside the saccade goal (Figs 3e,f and 4e,f). Unlike SCs neurons, SCi neurons did not encode visual saliency in the way described by several well-known computational models^1^,^15^,^16^. This observation echoes a recent study that examined saliency coding in the FEF during search in natural stationary images^33^. In that study, the authors concluded that FEF does not represent bottom-up saliency, but is more dominated by goal-directed selection and saccade planning processes. SCi has dominant inputs from FEF^20^, and plays an important role in visual attention and target selection^26^,^27^,^28^,^29^. Thus, SCi is more closely associated with the role of a priority map^4^, which plays an important role in determining which salient signals are selected for the moment-by-moment locus of gaze.

Computational modelling of visual saliency has had a profound impact on how we conceptualize the human visual attention system, and the development of artificial attention systems^1^. This study provides the first direct link between the discharge patterns of neurons in the midbrain SC elicited by dynamic natural scenes, and the output of a computational saliency model, built from the architecture of now well established models^1^,^15^,^16^, and validated on the viewing behaviour of humans and rhesus monkeys^31^,^32^. The saliency code observed in SCs is unlikely to be inherited directly from fronto-parietal cortices^10^,^11^,^12^,^13^,^14^, because those areas do not project to SCs. However, saliency may be computed in SCs and then relayed to other brain areas via tectothalamic pathways^34^,^35^,^36^. Future studies may benefit from the use of such a computational tool to make sense of visual activation patterns evoked by complex natural stimuli.

## Methods

### Subjects

Data were collected from three male Rhesus monkeys (*Macaca mulatta*) weighing between 10–12 kg. The surgical procedures and extracellular recording techniques have been detailed previously^37^, and were approved by the Queen's University Animal Care Committee in accordance with the guidelines of the Canadian Council on Animal Care.

### Stimuli and data acquisition

All stimuli were presented on a high-definition (HD) LCD video monitor (Sony Bravia 55′′, Model KDL-46XBR6) at a screen resolution of 1,920 × 1,080 pixels (60Hz non-interlaced, 24 bit colour depth, 8 bits per channel). Viewing distance was 70 cm resulting in a viewing angle of 82° horizontally and 52° vertically. The room was completely dark except for the illumination emitted by the monitor. The viewing area that extended beyond the monitor was blackened using black non-reflective cloth.

The tasks were controlled by a Dell 8100 computer running a UNIX-based real-time data control system (REX 7.6 (ref. 38)), which communicated with a second computer running in-house graphics software (written in C/C++) for presentation of stimuli. The HD video was displayed using a third Linux-based computer running custom software (downloadable at http://iLab.usc.edu/toolkit) under real-time kernel scheduling to guarantee accurate frame-rate^39^. Eye position was monitored using the scleral search coil technique^40^ in two animals, and a video-based eye tracker (Eyelink-1000, SR Research) in a third animal. The data were digitized and recorded in a fourth computer running a multi-channel data-acquisition system (Plexon Inc., Dallas, Texas, USA). Spike waveforms were sampled at 40 Khz. Eye position, event data, and spike times were digitized at 1 KHz.

The HD videos were converted into 40 Mbits per s MPEG-4 format (deinterlacing when required), and displayed at full resolution (1,920 × 1,080 pixels). The videos were obtained from commercial and in-house sources. Commercial sources included the BBC (British Broadcasting Corporation, London, UK) Planet Earth collection, BBC Wild Pacific, BBC Wild India, and several in-house collections filmed at locations in Los Angeles, CA, and Kingston, ON, with a HD camcorder (Canon Vixia HF S20). A total of 516 clips (102,161 distinct video frames), with durations ranging from 4 to 35 s, were extracted by parsing each video at jump points (points where the video abruptly changed scenes). Stimulus frame timing was confirmed using a photodiode placed at the left lower corner of the monitor and hidden by non-reflective tape. The photodiode measured an alternating white then black stimulus (20 × 20 pixels at bottom left of screen) on each frame of the video. The photodiode signal was recorded concurrently with spikes and eye position, and was used offline to recover the precise onset and duration of each video frame. Each video was randomly selected from the set. After all videos were viewed once by a given monkey, the set was allowed to repeat. There were 4,267 clip viewings in total across three monkeys over a period of approximately 5 months of data collection.

### Procedure

The animals were seated in a primate chair (Crist Instr., MD, USA) approximately 70 cm from the LCD video, head restrained. Tungsten microelectrodes (2.0 MΩ; Alpha Omega, Israel) were lowered into the SC. During this time the animals viewed a dynamic video, which provided rich visual stimulation that facilitated the localization of the visually-responsive dorsal SC surface. When a neuron was isolated, its visual RF was mapped using a rapid visual stimulation procedure described previously^41^. The animals then performed a delayed saccade task to characterize whether neurons had visual and/or motor responses, using previously established methods^23^,^29^,^41^. This was followed by the free-viewing task. Each HD clip was initiated by the monkey by fixating a central fixation point for a short period (500 ms). The animals were engaged in the dynamic video, so a reward protocol was generally not required for monkeys to initiate the clips. However, on some occasions, typically near the end of a session, a small liquid reward was given during the inter-clip interval to motivate the animals to continue initiating the clips. A typical session lasted 2–3 h.

*Calibration*. Each monkey performed a thorough 72-point calibration procedure. Briefly, the animals made saccades to a series of targets that spanned most of the screen (nine eccentricities, eight radial orientations). The targets appeared in random order, with multiple counts per location, and the animals were required to fixate the targets for a minimum of 300 ms for a liquid reward. The mode of the distribution of eye position points for each target was computed using the mean-shift algorithm^42^ with a bandwidth of 10 pixels (0.5°). If multiple clusters emerged, the cluster with maximum count was taken as the true eye position. If two clusters had the same count, the bandwidth was increased and the procedure repeated until a dominant cluster emerged or the maximum bandwidth of 15 pixels was reached, at which point the location was rejected. Eye position space was then corrected using an affine transformation with outlier rejection to recover the linear component of the transformation, and a thin-plate-spline to recover the nonlinear component^31^. In the third animal using the video-based eye tracker, we removed eye blinks offline by interpolating eye position before and after each blink. During the free-viewing task, the same mean-shift procedure described above was used for drift correction during central fixation just before clip onset. Saccades were defined as eye movements that exceeded a velocity criterion of 50° per s and a minimum amplitude of 1°.

*Neuron classification*. Single units were isolated online using a window discriminator, and confirmed offline using spike sorting software (Plexon Inc., Dallas, Texas, USA). A total of 82 SC neurons were isolated. Nine neurons were excluded because the isolation was lost after <20 clip viewings. Six neurons were excluded because of an unreliable photodiode signal required to synchronize the video with spike trains and behaviour. Six neurons were excluded because their RFs were too close to the fovea (<2° eccentricity) for the saccade direction analyses. Two neurons were excluded because they were not visually responsive. The remaining 60 neurons formed the basis of the analysis (*n*=27 from monkey 1; *n*=12 from monkey 2; *n*=21 from monkey 3). Spikes were convolved with a function that resembled an excitatory post-synaptic potential^43^, with rise and decay values of 5 and 20 ms, respectively.

The SC is comprised of two dominant functional layers^20^,^44^, a visual-only superficial layer (SCs), and a multisensory/cognitive/motor-related intermediate layer (SCi). The neurons were functionally classified as visual-SCs or visuomotor-SCi based on their discharge characteristics using a visual RF mapping procedure^41^ to determine the presence of a visual component, and a delayed-saccade task to determine the presence of a motor component, using previously established methods^23^,^29^,^41^. Briefly, neurons were defined as having a visual component if the visual mapping procedure yielded a localized hotspot^41^. We also confirmed visual responses of most (51/60) neurons using the delayed saccade task, by determining whether the average activity immediately following stimulus onset (40 to 120 ms post stimulus) was significantly greater than the average activation over a pre-stimulus baseline period (−80 ms to stimulus onset). Neurons were defined as having a motor component if the average firing rate around the time of the saccade (−25 to +25 ms relative to saccade onset) was significantly greater than a pre-saccadic baseline period (−150 to −50 ms relative to saccade onset). For nine neurons we were unable to obtain data from the delayed saccade task, so we estimated their motor-related discharge during free-viewing using the same criteria above on a subset of the saccades that were directed into the RF. In total, 26 neurons were classified as visuomotor SCi, and the remaining 34 were classified as visual SCs. Of the 34 neurons classified as SCs, the majority of these (29, 85%) were estimated to be sampled within 1 mm of the dorsal surface of the SC. The remaining five were estimated to be deeper. The results were qualitatively similar between these five neurons and the other 29 labelled as SCs. Also, the statistical results were the same with or without these five neurons, so we combined them in the final analysis.

*Fixation durations and epochs*. Ninety-five percent of fixation durations fell between 95 and 545 ms (Supplementary Fig. 1). A minimum fixation duration cutoff of 200 ms was chosen to allow for a sufficient saccade-free, visual integration period while retaining a sufficient amount of data (50,659 total fixations). Averaging was computed over the pre-saccadic period (0–200 ms).

*Data normalization*. Neuronal discharge rates were normalized for each neuron using a 0-1 rescaling of the spike density function via the following equation,


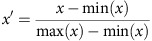


where *x*′ refers to the normalized spike density function for a given condition, and *x* refers to the original spike density function for that condition. Min(*x*) and max(*x*) refer to the minimum and maximum values within the post-fixation period (0–500 ms).

*Statistics*. The main statistical analysis was a repeated-measures ANOVA, whose assumptions were met for use in this study: (1) The independent variables (saliency-level and saccade-goal) were repeated/matched (that is, saliency-level and saccade-goal were extracted from within the same experimental sessions). (2) The dependent variable (neuronal discharge rate) was continuous. (3) The data across all conditions met the normality assumption based on Kolmogorov–Smirnov test (*P*<0.05). (4) The error variance between conditions was equal (that is, Mauchly's sphericity test of equal variances was not violated). Observed statistical power was greater than 0.7 for the main interaction between saliency-level and saccade-goal, and greater than 0.93 for all other significant main effects. *T*-tests were one-tailed unless otherwise stated, and based on *a priori* directional hypotheses. Alpha levels for multiple *t*-tests were Bonferroni-corrected, and running statistical tests were corrected using Bonferroni-Holm method for time-series data.

**Figure 2:**
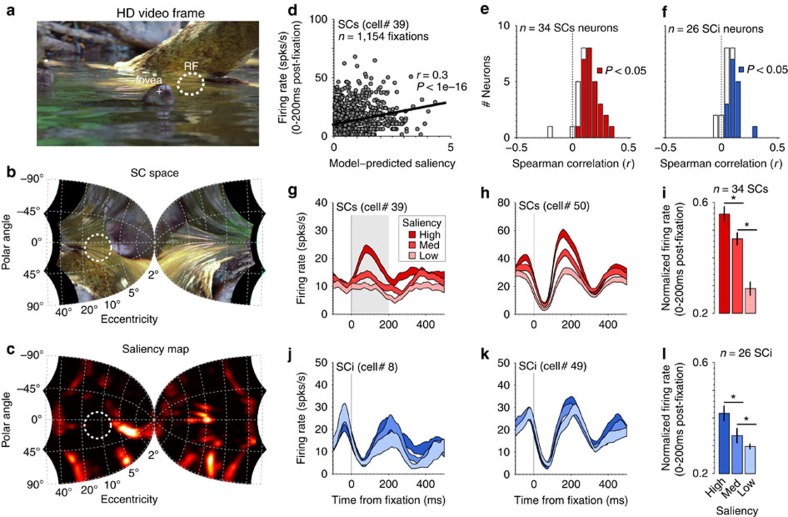
Saliency coding in the SC during free viewing of natural dynamic scenes. (**a**) Single frame of an HD clip (crosshair: eye position; annulus: receptive field (RF) of the neuron). (**b**) Transformation into log-polar SC space based on^24^. (**c**) Model-predicted pattern of activation across the SC map. The black regions in **b** represent the viewing area that extended beyond the monitor, and was blackened using non-reflective cloth (see Methods). The annulus in **b** and **c** represents the approximate point image corresponding to the RF in **a**. (**d**) Spearman correlation between model-predicted saliency and firing rate of a single SCs neuron. (**e**,**f**) Distributions of r-values for the correlation between model-predicted saliency and firing rate for the sample of 34 SCs neurons (**e**), and 26 SCi neurons (**f**). (**g**,**h**) Average firing rate (±1 standard error of the mean; s.e.m.) of two example SCs neurons as a function of time from fixation onset, when the saliency values in the RF were divided into tertiles (low, medium, high). Only fixations with duration >200 ms were included. (**i**) Average normalized firing rate of the 34 SCs neurons during the saccade-free epoch (0–200 ms post fixation) illustrated by the grey shaded region in **g**. (**j**,**k**) Average firing rate (±1 s.e.m.) of two example SCi neurons as a function of time from fixation onset, for the three saliency levels. (**l**) Average normalized firing rate of the 26 SCi neurons during the saccade-free epoch (0–200 ms post-fixation). Error bars in **i** and **l** indicate ±1 s.e.m. *P<0.05, paired *t*-test, one-tailed.

**Figure 3:**
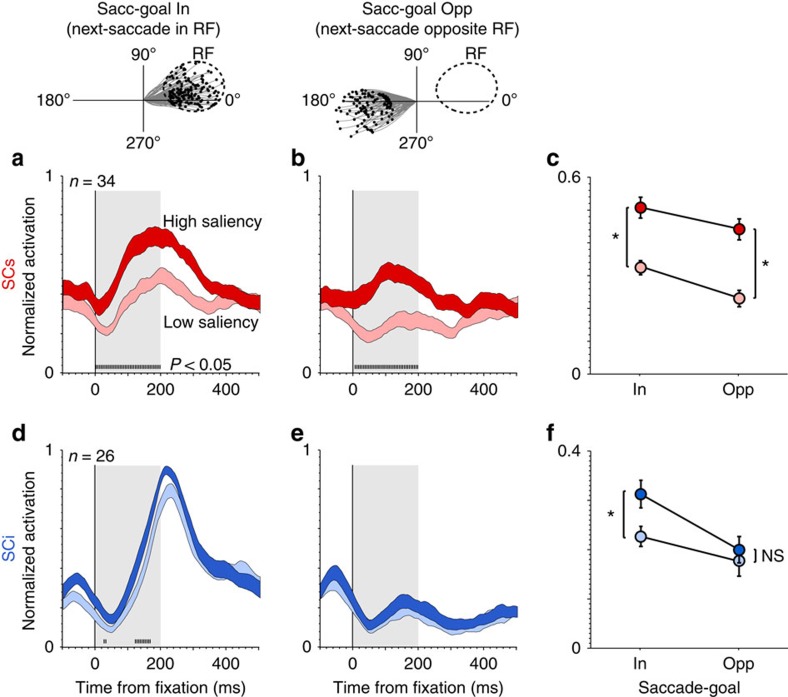
Behavioural dependence of SC saliency coding (fixation-aligned). A subset of the total fixations were extracted based on saccade-goal (that is, the direction of the next-saccade relative to the RF; top illustration). (**a**,**b**) Average normalized firing rate of SCs neurons associated with high (dark red) versus low (light red) saliency (upper versus lower tertiles), when next-saccades were directed in **a** versus **b** opposite the RF. (**d**,**e**) Average normalized firing rate of SCi neurons associated with high (dark blue) versus low (light blue) saliency, when next saccades were directed in **d** versus **e** opposite the RF. Shading along the response curves indicates ±1 s.e.m. Tick marks above the abscissa indicate significant differences between the response curves within the pre-saccadic epoch (5 ms bins, Wilcoxon paired-samples test, Bonferroni-Holm correction). The dotted vertical line in **a**,**b** and **d** indicates the time the response curves first differed. (**c,f**) Average normalized firing rate during the pre-saccadic epoch (0–200 ms) illustrated by the gray shaded regions in **a**,**b**,**d** and **e**. Error bars in **c** and **f** indicate ±1 s.e.m. *P<0.05, paired *t*-test, 1-tailed; NS=not statistically significant.

**Figure 4:**
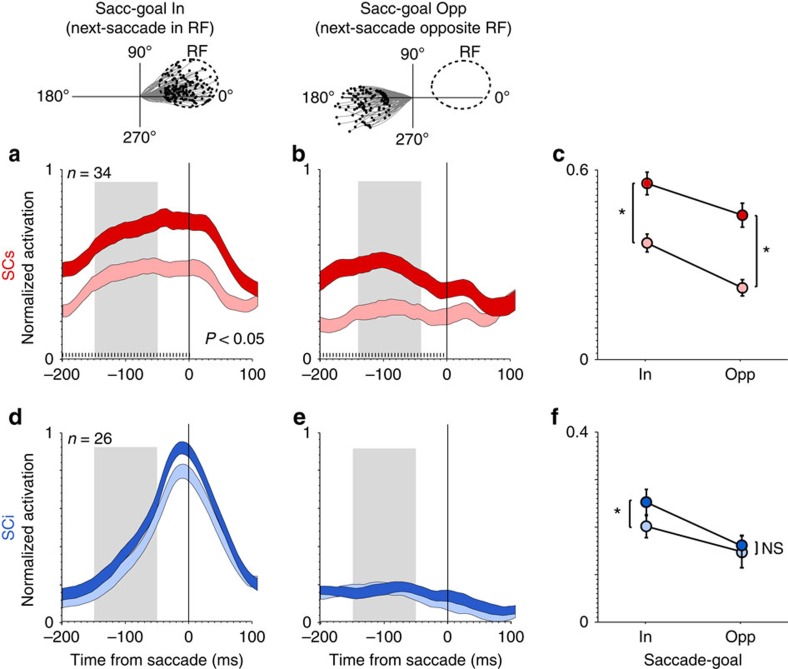
Behavioural dependence of SC saliency coding (saccade-aligned). (**a,b**) Average normalized activation of SCs neurons associated with high (dark red) versus low (light red) saliency (upper versus lower tertiles), when next-saccades were directed in **a** versus **b** opposite the RF. (**d**,**e**) Average normalized activation of SCi neurons associated with high (dark blue) versus low (light blue) saliency, when next saccades were directed in **d** versus **e** opposite the RF. Shading along the response curves indicates ±1 s.e.m. Tick marks above the abscissa indicate significant differences between the response curves (5 ms bins, Wilcoxon paired-samples test, Bonferroni-Holm correction). (**c**,**f**) Average normalized activation within a pre-saccadic epoch (−150 to −50 ms) illustrated by the grey shaded regions in **a**,**b**,**d** and **e**). Error bars in **c** and **f** indicate ±1 s.e.m. *P<0.05, paired *t*-test; NS=not statistically significant.

**Figure 5:**
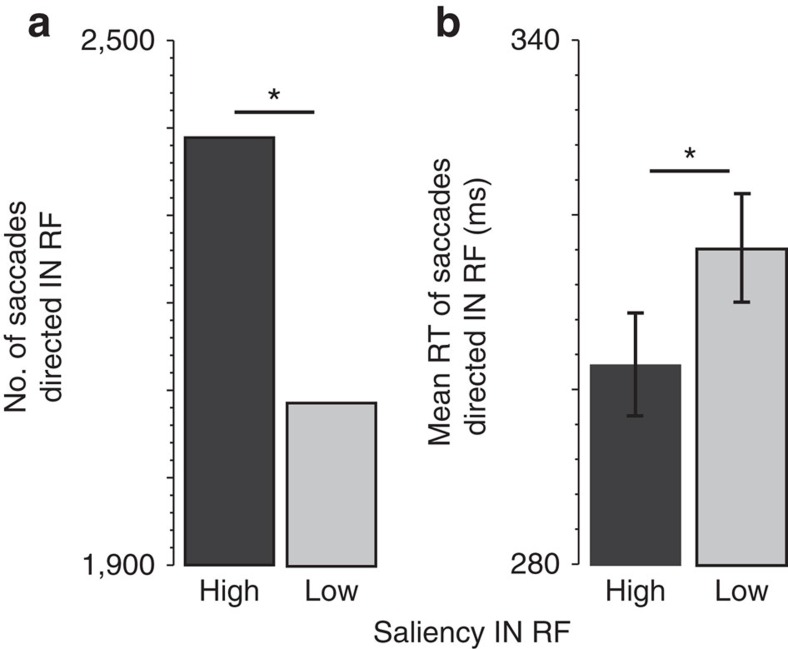
Model saliency predicts saccade behaviour. (**a**) Number of saccades directed IN the RF as a function of model-predicted saliency. (**b**) Mean saccade reaction time (RT; that is, fixation duration) for saccades directed IN the RF as a function of model-predicted saliency. *P<0.05, Wilcoxon independent-samples test.

**Figure 6:**
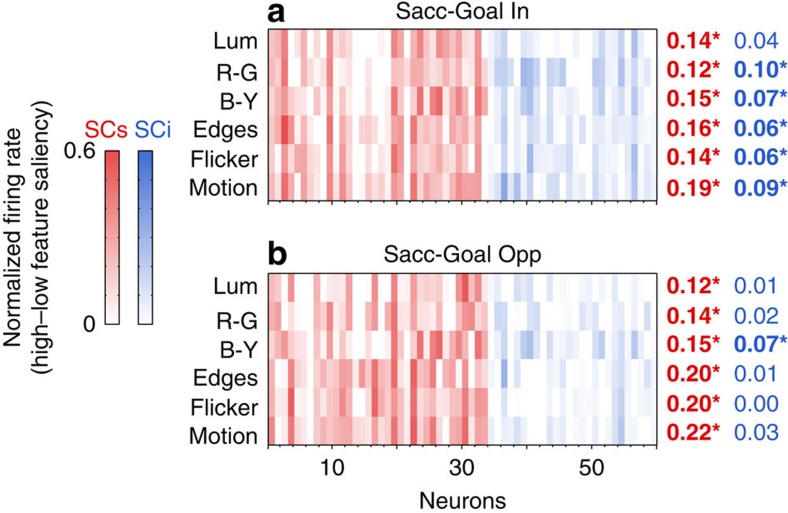
Feature dependence of SC saliency coding. Feature-specific activation profiles of SCs (red; *n*=34) and SCi (blue; *n*=26) neurons when the saccade-goal was in (**a**) versus opposite (**b**) the RF. Each column represents a given neuron, and the intensity of the colour represents the difference in activation evoked by high versus low model-predicted feature contrast (colour scale on the left). The table of numbers on the right indicates the mean across neurons for a given feature, with the bolded numbers/asterisk indicating the significant features. *P<0.05, Bonferroni-corrected *t*-tests, 1-tailed. Abbreviations: Lum: luminance; R–G: red–green colour opponency; B–Y: blue–yellow colour opponency.

*Model overview*. The general architecture of the saliency model has been described in detail^1^,^15^,^16^, and was created and run under Linux using the iLab C++Neuromorphic Vision Toolkit^45^. Briefly, the model is feed-forward in nature and consists of six high-level feature maps that are coarsely tuned to the primate visual system^32^: luminance centre-surround contrast, red-green opponency, blue-yellow opponency, orientation/edges, flicker centre-surround (abrupt onsets and offsets), and motion centre-surround (responding to local motion that differed from surrounding full-field motion) (Fig. 1a). The high-level feature maps were linearly combined with equal weighting to create a feature-agnostic saliency map. The feature and saliency maps were computed in a gaze-contingent manner (each video frame was shifted to fovea-centred coordinates). The model was computed on a log-polar transformation of the input image (Fig. 2) to approximate the non-homogeneous mapping of visual- to SC space^24^. Saliency within a neuron's RF was computed as the normalized sum of the saliency output within the region specified by the RF.

*Retinal input*. Each HD video frame was first shifted to retinal coordinates (that is, the centre of gaze was always the centre of the input to the model), replacing any empty values with black to match the viewing environment beyond the screen. Each frame was embedded in a larger black background image to simulate 100° × 100° of the viewing environment (the size of our SC saliency map). The eye movement data were down-sampled from 1,000 Hz to 200 Hz, and the video was processed at a matched 200 Hz to accurately capture the visual dynamics associated with rapid eye movements. Thus, each eye position sample gave rise to a new retinal image, even when the video display was unchanged. The retinal image was rescaled by decimating and smoothing with a 3-tap binomial filter, and converted to the Derrington-Krauskopf-Lennie (DKL) colour space^46^ to approximate the luminance, red-green opponent, and blue-yellow opponent systems in early vision.

*Space-variant transformation*. The primate retinostriate and retinotectal projections create a nonhomogeneous mapping of visual space such that most of the neural surface is dedicated to foveal processing. In the SC, this has been described as a log-polar transformation^24^, logarithmic with respect to eccentricity from the fovea, and polar with respect to angular deviation from the horizontal meridian. One disadvantage of this mapping is that it is discontinuous at the vertical meridian, which makes standard image processing techniques unusable. Instead of a conformational mapping to the SC surface, we performed a simpler log-polar resampling of the input image which captures key features of the retinotectal mapping^47^. The result of the transform was a square image on which standard image processing can be applied.

Model SC units were simulated on a two dimensional (200 × 200) grid representing approximately 100° × 100° of visual angle. To compute the point in visual space (or image space) corresponding to the RF centre of each SC unit, we used the inverse of the basic variable resolution transform^47^. The scaling parameters of the transform were set by simulating a square grid of SC surface (4.5 mm rostral-caudal and 3.5 mm medial-temporal for each hemifield) and using the inverse conformational mapping^24^ to find the corresponding points in visual space. The SC model map points were also projected to visual coordinates using the inverse basic variable resolution transform, and a least squares fit, minimized with a simplex-algorithm using 1,000 random restarts^48^, resulted in the best scaling parameters.

*Raw feature computation*. The model's six high-level feature maps in SC space were created by linearly combining the responses of different filters (low-level feature maps) of the same type (for example, 45° and 90° edge detectors). In total, there were 60 filters and associated low-level feature maps: one each for luminance, red-green, and blue-yellow opponent contrasts, 8 for static oriented edges computed at 4 orientations and 2 spatial scales (retinal and half retinal), one for flicker, and 48 for opponent motion computed by pair-wise subtraction from 24 raw motion maps computed at 3 speeds, 4 orientations and 2 spatial scales. All features were computed in a centre-surround architecture and thus signaled salient local differences between centre and surround regions (see below). To compute the orientation, flicker and motion maps, which were all derived from luminance in the DKL space, the luminance images were buffered for 9 frames and processed by a three-dimensional 2nd derivative of Gaussian separable steerable filter^49^. The steerable set allows for the creation of the required 57 spatio-temporal filters as linear combinations of base filters from a small basis set. The filters were computed in quadrature pair, and the magnitude was taken as the filter response. Hence, the filters responded to both step edges and bars (static, flickering or moving), as in models of V1 complex cells^50^. Raw feature maps described here were further endowed with non-linear horizontal connections that further emphasized salient regions in each map (described below).

*Modelling SC receptive-fields*. SC Neurons are reported to have a centre-surround receptive field structure such that a disk stimulus larger than a preferred size begins to inhibit responses^25^. Preferred stimulus size ranges from 0.75° near the fovea to approximately 5° at 40° eccentricity^51^; however, SC neurons have large response fields of 10°–40° diameter that are largely invariant to the exact position of the stimulus within the RF^52^. The size specificity with large invariant activation fields suggests a simple model where the SC pools responses of Difference-of-Gaussian (DoG) detectors at nearby spatial locations.

Receptive fields were modelled by first computing a Gaussian scale space^53^. A DoG detector was built by subtracting a sample at a lower level from one at the same spatial location at a higher level in the scale space. The spatial locations of the samples are given by the space-variant transform. The level (receptive field size) was computed from previously established estimations^51^. To create a DoG detector sensitive to an optimal stimulus size S in degrees, and DoG ratio K, the excitatory Gaussian size in degrees was,


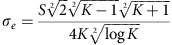


and the inhibitory size was,


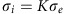


DoG receptive fields were constructed linearly with eccentricity with an optimal stimulus size of 0.75° at the fovea, and 5° at 40° eccentricity. The space-variant remapping and centre-surround receptive fields were computed for each feature map. For the luminance and chromatic features, we used *K*=6.7 based on experiments in retinal ganglion cells^54^. For these features, the absolute value of the DoG response was taken giving rise to double opponency responses (for example, responds to red surrounded by green and green surrounded by red). For the spatiotemporal feature maps, *K*=3.2 was used based on studies in V1 (ref. 55) and the DoG responses were half-wave rectified. To model long-range competition in the SC^25^ a single iteration of the salience competition operator of Itti and Koch^56^ was applied to each feature map. Briefly, the map was first filtered with a large DoG (3° excitatory, 9° inhibitory). The result was added back to the feature map and a constant subtracted to represent global inhibition, followed by a final half-wave rectification. This operator has been argued to capture some of the non-classical surround effects in early visual processing^56^. Each point in each 200 × 200 map was then replaced by the sum in a 3 pixel circular neighbourhood to simulate the large (but size selective) activation fields observed in the SC. This resulted in model activation fields which were approximately 2° at 10° eccentricity and 10° at 40° eccentricity, similar to those in the SCs^51^. Due to pooling and symmetrically filtering in the SC model space, the activation fields also have an asymmetry such that they slightly narrow toward the fovea, as described elsewhere^37^.

For the saccade-in/opposite analysis, which used only a subset of the total saccades/fixations, a less conservative estimate of the RF boundary was used for inclusion of saccades/fixations. We estimated the RF boundary of each neuron by converting the boundary of a circular point image in SC space to visual space using the reverse mapping equations from Ottes *et al*.^24^


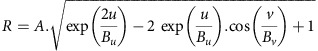



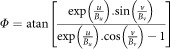


where *R* and *Φ* represent the eccentricity and polar angle of points along the RF boundary. *u* and *v* represent the horizontal and vertical position of points along the point image boundary. *A*, *B*_*u*_, and *B*_*v*_, whose values were 3, 1.4 and 1.8 respectively, are constants that determine the precise shape of the mapping. We chose a point image diameter of 1.5 mm (based on (ref. 37)), which was centred on the SC map location that corresponded to each neuron's optimal visual RF centre (derived from the course visual mapping procedure described earlier).

*Data fitting and saliency response*. For every neuron and video in the analysis, 60 gaze-centred, space-variant feature maps were computed at 200 frames per s, and values were collected over the duration of each video at the location corresponding to the cell's receptive field centre (obtained from the receptive field mapping paradigm). Spike trains during each clip were binned (5 ms intervals) and convolved with a 50 ms Gaussian filter. The data fitting took place for each cell separately using a leave-one-out training such that each clip was excluded from the set once for testing, and the model was trained on all the rest. The collected feature values in the test clip were normalized by the maximum in the training set (across all clips for each feature separately) and linearly combined into the six high-level features. Before combining the high-level features into the saliency response, each feature was optimally aligned to the spike density function. The optimal delay for each feature was computed by calculating the mutual information at different time delays (up to 150 ms) between test-set features and test-set neural responses, considering all clips. After optimal alignment, the square root of each high-level feature was taken and the responses were linearly combined to create the saliency response.

### Code availability

The Computer Code for the saliency model is available from coauthor Laurent Itti (University of Southern California, CA, USA) upon reasonable request.

### Data availability

The data that support the findings of this study are available from the corresponding author upon reasonable request.

## Additional information

**How to cite this article:** White, B. J. *et al*. Superior colliculus neurons encode a visual saliency map during free viewing of natural dynamic video. *Nat. Commun.* **8,** 14263 doi: 10.1038/ncomms14263 (2017).

**Publisher's note:** Springer Nature remains neutral with regard to jurisdictional claims in published maps and institutional affiliations.

## Supplementary Material

Supplementary Information Supplementary Figures
