# Supplementary material for: Superior colliculus neurons encode a visual saliency map during free viewing of natural dynamic video
Source: Nat Commun. 2017 Jan 24;8:14263. doi: 10.1038/ncomms14263 (PMC5286207; doi:10.1038/ncomms14263)
Supplement: Supplementary Information — Supplementary Figures [file ncomms14263-s1.pdf]

Supplementary Figures

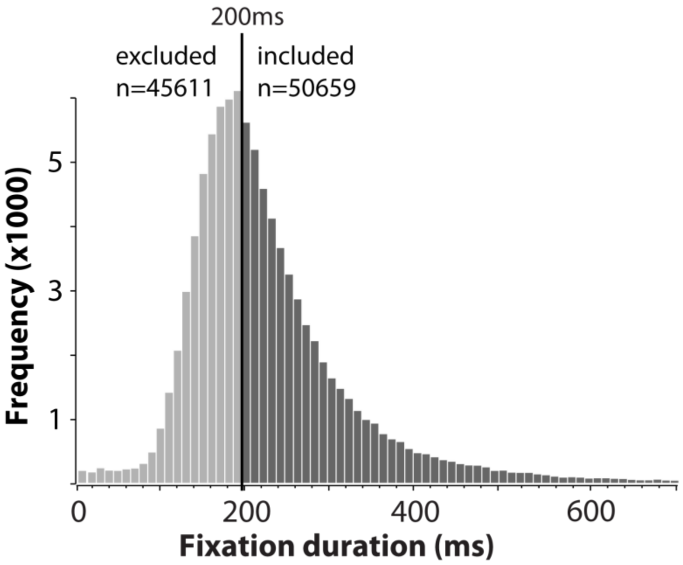

Supplementary Figure 1: Total distribution of fixation durations across all recording sessions. Dark bars indicate the fixations whose duration exceeded the 200ms cutoff.

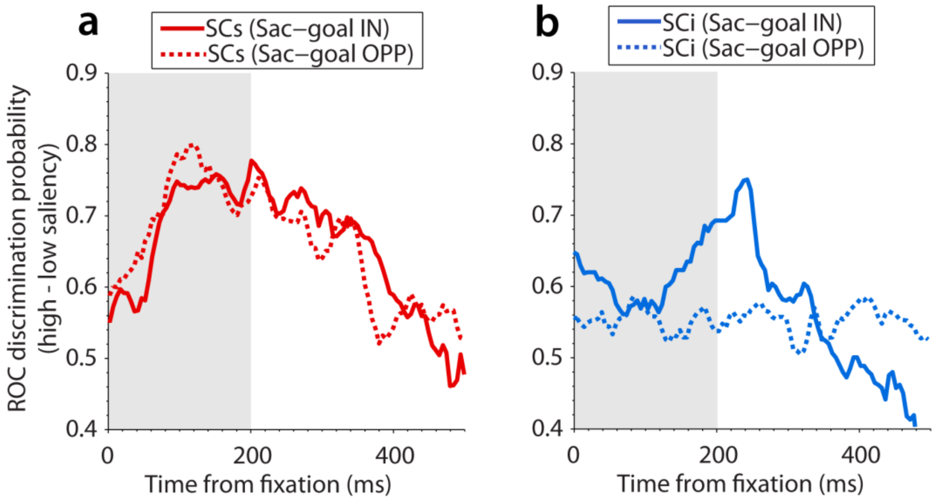

<u>Supplementary Figure 2.</u> Receiver operating characteristic (ROC) analysis. We computed the area under the ROC curve at each millisecond, between the high-saliency and low-saliency conditions, across the sample of **(a)** SCs (n= 34), and **(b)** SCi (n= 26) neurons, separately for the saccade-goal In (solid traces) and saccade-goal-opposite (dotted traces) conditions. Values greater than 0.5 indicate the neurons' ability to discriminate high from low saliency (i.e., greater firing rate associated with high saliency).

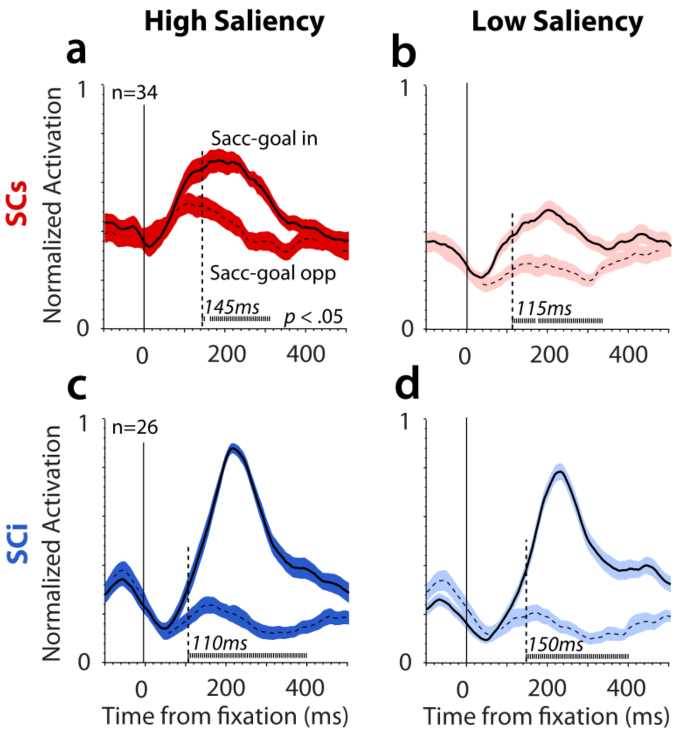

Supplementary Figure 3. **Selection processes during free viewing.** The selection process of SCs and SCi was quantified during free viewing by comparing activation associated with next-saccades directed in (solid lines) versus opposite (dashed lines) the RF. Shading along the response curves represents ±1 SEM. Tick marks above the abscissa in indicate significant differences between the response curves (running Wilcoxon paired-samples test, Bonferroni-Holm correction). Selection time (the earliest point where the response curves significantly diverged) is indicated by the vertical dotted lines.
